# APP-based treatment of urgency and mixed urinary incontinence in women: factors associated with long-term satisfaction

**DOI:** 10.1007/s00404-023-07303-2

**Published:** 2023-12-23

**Authors:** Towe Wadensten, Emma Nyström, Malin Sjöström, Anna Lindam, Eva Samuelsson

**Affiliations:** 1 Department of Public Health and Clinical Medicine, Umeå University, 901 87 Umeå, Sweden; 2 Unit of Research, Education, and Development, Östersund Hospital, Östersund, Sweden

**Keywords:** eHealth, Mobile app, Treatment satisfaction, Urgency urinary incontinence, OAB

## Abstract

**Purpose:**

App-based treatment of urgency (UUI) and mixed (MUI) urinary incontinence has proved to be effective. To further improve treatment, it will be beneficial to analyze baseline and treatment-related factors that are associated with satisfaction.

**Methods:**

A secondary analysis was conducted of data from a randomized controlled trial (RCT) assessing an app for UUI or MUI treatment, encompassing 98 women for whom there was long-term treatment satisfaction data. All participants completed a short-term (15 weeks) and a long-term (15 months) follow-up questionnaire after being given access to treatment. The outcome was a 3-item question on current treatment satisfaction at the long-term follow-up. Factors potentially associated with the outcome were analyzed using the chi-square test, Student’s *t* test or logistic regression.

**Results:**

At the long-term follow-up, 58% of the women were satisfied with the treatment. The most important baseline variable associated with satisfaction was incontinence-related quality of life (International Consultation on Incontinence Questionnaire (ICIQ) − Lower Urinary Tract Symptoms Quality of Life Module) (OR 0.91, 95% CI 0.58–0.97). Short-term follow-up variables associated with long-term treatment satisfaction were improvement in the ability to endure urgency (OR 4.33, 95% CI 1.43–13.12), and confidence in pelvic floor contraction ability (OR 2.67, 95% CI 1.04–6.82).

**Conclusion:**

App-based treatment for UUI and MUI may be an alternative first-line treatment that is satisfactory to many women over the long-term. Furthermore, short-term treatment that focuses on improving the ability to endure urgency, and confidence in pelvic floor contraction ability, can also be recommended for long-term satisfaction.

## What does this study add to the clinical work?

**Table:** 

A mobile app treatment for female urgency and mixed urinary incontinence was associated with a high rate of satisfaction in the long term, especially among users with a lower impact on quality of life at baseline.	
An increased ability to endure urgency and an improved pelvic floor contraction ability were also associated with treatment satisfaction.	

## Introduction

Urgency (UUI) and mixed (MUI) urinary incontinence are common conditions among women and often have a negative effect on quality of life [1–3]. The recommended first-line treatment includes pelvic floor muscle training (PFMT) and lifestyle interventions, sometimes combined with bladder training [1, 4]. We have previously demonstrated that a mobile app was effective for the self-management of UUI and MUI both over the short term and long term [5, 6]. Knowledge of the factors connected to a satisfactory treatment result could aid the clinician in guiding patients to the most suitable treatment options from the beginning. Furthermore, a better understanding of important elements of e-health treatment could provide valuable input for developing and improving e-health interventions.

The aim of this study was, therefore, to investigate factors at baseline and during the treatment period that are associated with a long-term satisfactory outcome.

## Methods

### Procedure

This study was a secondary analysis of data from a randomized controlled trial (RCT) evaluating the app Tät^®^II (Clinicaltrials.gov, NCT03097549). The procedure included monitoring for proper research conduct, as described in detail elsewhere [5].

**Fig. 1:**
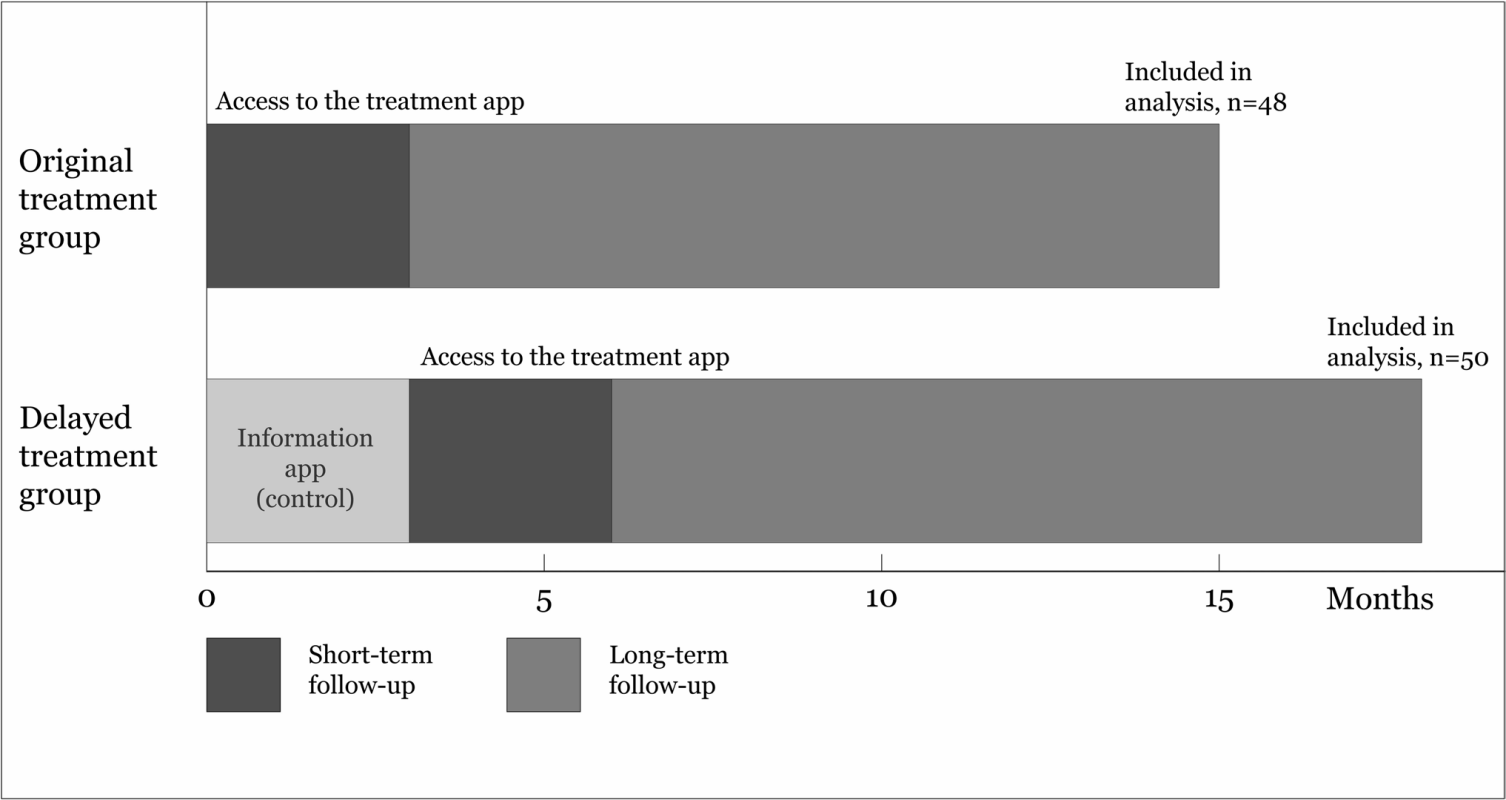
Overview of the study flow. In this study, the short-term follow-up was the second follow-up for the delayed treatment group; the first follow-up for this group was conducted before the group received access to the treatment app. For both groups, the duration of the app treatment was 15 weeks, followed by maintenance pelvic floor muscle training. The long-term follow-up was performed 12 months after the 15-week treatment ended. In the current study, the two groups were analyzed as one

Participants in the RCT were randomized to a treatment app group or an information app group (control). After 15 weeks, both groups submitted follow-up questionnaires and bladder diaries. Participants in the information app group were then given access to the treatment app and underwent another follow-up procedure after 15 weeks. In the current study, the data collection after 15 weeks of access to the treatment app is referred to as “the short-term follow-up”. Both groups received a questionnaire 15 months after being given access to the treatment app, i.e., “the long-term follow-up” (Fig. 1).

The current study was approved by the Regional Ethical Review Board of Umeå, Sweden and the Swedish National Ethical Review Board (registry numbers 2016/523–31 and 2021–06772-02). All participants provided written informed consent.

### Inclusion/exclusion criteria

The original RCT study included 123 women ≥ 18 years old, with UUI or MUI and ≥ 2 urinary leakages per week. Exclusion criteria were: pregnancy; incontinence-related medication; neurological conditions; diabetes; and cancer in the pelvic area. Individuals expressing red-flag symptoms (painful urgency; previous pyelonephritis; repeated urinary tract infections; dysuria; visible hematuria; bladder-emptying difficulties; and metrorrhagia) were excluded and encouraged to contact their healthcare practitioner.

The current study included all participants for whom there was data on treatment satisfaction at the long-term follow-up (*n* = 98).

### Intervention

The app Tät^®^II includes PFMT and bladder training; psychoeducation and lifestyle advice; exercise statistics; tailored advice; automated feedback; and reminders. Users are recommended a more intense training regime (3 times per day) for the first 15 weeks of using the app, followed by less frequent maintenance training (2–3 times per week). The app was developed by ES and TW, in collaboration with other researchers and the technical development division (ITS) at Umeå University. A detailed report has previously been published [5].

### Data collected

The data collected at baseline included the following validated questionnaires: the International Consultation on Incontinence Questionnaire (ICIQ) − Urinary Incontinence Short Form (ICIQ-UI SF) (assessing urinary leakage, 0–21 points); the ICIQ − Lower Urinary Tract Symptoms Quality of Life Module (ICIQ-LUTSqol) (assessing incontinence-related quality of life, 19–76 points); and the ICIQ − Overactive Bladder Module (ICIQ-OAB) (assessing urgency symptoms, 0–16 points). For all these scales, a higher score indicates a greater level of impact of the incontinence [7]. Data on self-perceived ability to perform a PFMT contraction; previous incontinence-related healthcare contact; the presence of other chronic conditions (i.e., hypertension; heart conditions; asthma; depression or anxiety; renal disease; cancer; or other); and current medications (i.e., medicines for heart conditions or hypertension, asthma, depression or anxiety; diuretics; oral estrogen; and hormonal intrauterine devices) was also collected, as well as data on age, body mass index (BMI), education level, geography, and occupation. The eHealth Literacy Scale (eHEALS) assessed the participants’ digital competence (8 items, 8–40 points) [8]. Additionally, participants rated their level of motivation and confidence in their ability to follow-through with the treatment program on a scale of 0–10 (higher numbers indicating higher motivation/confidence), and their expectations regarding the treatment results (a little improved; much improved; very much improved; or completely rid of leakage).

In both the short-term and long-term follow-up questionnaires, the questions on incontinence symptoms, BMI data, medication, and incontinence-related healthcare contact were repeated. In addition, questions on app usage and training frequency; ability to perform correct PFMT contractions and endure urgency; and level of motivation for maintenance training, were included. The Patient Global Impression of Improvement (PGI-I) assessed both short-term and long-term improvement, with seven response options ranging from “Very much better” to “Very much worse” [9].

### Treatment satisfaction

Long-term satisfaction with the treatment was assessed using the question “Do you currently consider that the treatment you have undergone is satisfactory?” at the 15-month follow-up, with three response options (“yes, I am completely rid of leakage and urgency”; “yes, I am not completely rid of leakage and urgency but still consider it satisfactory”; or “no”).

### Factors potentially associated with treatment satisfaction

#### Baseline

The categorical variables considered for inclusion in this model were randomization group; BMI category; constipation; education; incontinence-related healthcare contacts; treatment expectations; other chronic conditions; concomitant medication; and type of incontinence.

The continuous variables included age; ICIQ-UI SF; ICIQ-LUTSqol; ICIQ-OAB; and eHEALS.

#### Short-term follow-up

The categorical variables considered for inclusion in this model were treatment satisfaction; PGI-I; app usage frequency; PFMT frequency; confidence in ability to perform maintenance training; confidence in ability to perform PFMT contractions correctly; change in pelvic floor contraction ability compared with baseline; bladder training frequency; and change in ability to endure urgency compared with baseline.

The continuous variables included the calculated difference between the follow-up score and the baseline score for the ICIQ-UI SF; ICIQ-LUTSqol; and ICIQ-OAB.

### Statistical analysis

**Fig. 2:**
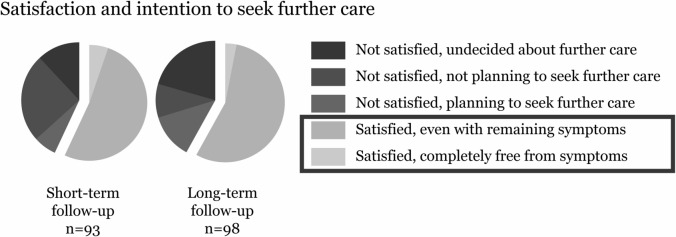
Overview of the outcome variable *treatment satisfaction*. The question regarding treatment satisfaction was included in both the short-term and long-term follow-up questionnaires. For participants who were not satisfied, a second question assessed their intent to seek additional care for their symptoms. In this study, a “satisfied” outcome was defined as treatment satisfaction with or without remaining symptoms

The long-term treatment satisfaction was dichotomized into “satisfied” (i.e., participants answering “yes”: value = 1) and “not satisfied” (i.e., participants answering “no”: value = 0) (Fig. 2), and used as the outcome variable.

First, each variable was analyzed separately against the outcome, using chi-square tests (categorical variables) or a Student’s *t* test (continuous variables). We then performed crude logistic regression analyses where variables with a *p* value of ≤ 0.2 were considered for further investigation. The final decision on the variables to include in the multivariable logistic regression models was guided by the results from the crude analysis and a discussion of their clinical relevance.

The selected variables were analyzed against treatment satisfaction in two separate multivariable logistic regression models: one model with baseline variables, and one model with short-term follow-up variables. With a sample size of 98 women, a maximum of 5 factors were allowed in each model to set a minimum of around 20 observations per variable.

To examine the relevance of treatment satisfaction as an outcome variable, we also compared the “satisfied” and the “not satisfied” group looking at the change in score for the ICIQ-UI SF; ICIQ-LUTSqol; and ICIQ-OAB from baseline to long-term follow-up, using a Student’s *t*-test.

SPSS versions 27 and 28 were used for the analyses.

## Results

### General information

A total of 98 women answered the question on treatment satisfaction at 15 months and were included.

The mean age was 58 years and just over half of the women were overweight or obese. A little less than half had concomitant chronic conditions. The majority had a university education and a high level of e-health literacy. Very few were smokers and most of the participants had a low or moderate coffee and tea consumption. Around three quarters of the women had MUI. The majority had their incontinence condition for a long time and most participants had not contacted healthcare services for their symptoms. The women had three urinary leakages per day on average (Table 1).

**Table 1:** Baseline variables

Baseline factors			
General		All participants	Range
Age, mean (SD)		58 (9)	37–77
BMI category, *n* (%)	Overweight	36 (37)	
	Obesity	21 (21)	
Low level of physical activity, *n* (%)^a^		51 (52)	
Smoker (weekly), *n* (%)^b^		2 (2)	
Daily coffee consumption ≥ 3 cups, *n* (%)		58 (59)	
Daily tea consumption ≥ 3 cups, *n* (%)		12 (12)	
University education, *n* (%)^c^		84 (86)	
eHEALS, mean (SD)^d^		33 (5)	13–40

Health			
Other chronic conditions, *n* (%)^e^		40 (41)	
Concomitant medications, *n* (%)^f^	Heart condition or hypertension	16 (16)	
	Asthma	7 (7)	
	Depression or anxiety	11 (11)	
	Oral estrogens	1 (1)	
	Hormonal intrauterine device	6 (6)	
	Diuretics	0 (0)	
	None﻿	62 (63)	
Constipation, *n* (%)^g^		25 (26)	

Gynaecology			
Never given birth, *n* (%)		11 (11)	
Prolapse symptoms, *n* (%)		7 (7)	
Postmenopausal, *n* (%)		68 (69)	

Incontinence			
Type of incontinence, *n* (%)	UUI	25 (26)	
	MUI	73 (74)	
Symptom duration > 5 years, *n* (%)		59 (60)	
No previous incontinence-related healthcare contact, *n* (%)		67 (68)	
Daily use of incontinence protection pads, *n* (%)		39 (40)	
ICIQ-UI SF, mean (SD)^h^		11.5 (3.3)	4–20
ICIQ-LUTSqol, mean (SD)^h^		37.8 (7.9)	23–60
ICIQ-OAB, mean (SD)^h^		6.7 (1.8)	3–13
QUID, mean (SD)^h^	Stress	5.9 (3.8)	0–15
	Urge	7.2 (2.3)	2–13
Incontinence episode frequency, mean (SD)^i^		21.2 (15.2)	0–77

Other			
Delayed access to treatment app, *n* (%)^j^		50 (51)	
Confident about PFMT contraction, *n* (%)^k^		38 (39)	
Expectations on treatment outcome, *n* (%)	Slightly or much improved	46 (47)	
	Very much improved or completely free from leakage	52 (53)	
Motivation to follow-through with the 15-week treatment program^l^	Mean (SD)	9.2 (1.0)	6–10
	Median (IQR)	10 (8.75–10.0)	

*BMI* Body Mass Index, *eHEALS* eHealth Literacy Scale, *UUI* Urgency urinary incontinence, *MUI* Mixed urinary incontinence, *ICIQ-UI SF* International Consultation on Incontinence Questionnaire (ICIQ) − Urinary Incontinence Short Form, *ICIQ-LUTSqol* ICIQ − Lower Urinary Tract Symptoms Quality of Life Module, *ICIQ-OAB* ICIQ − Overactive Bladder Module, *QUID* Questionnaire for Urinary Incontinence Diagnosis, *PFMT* Pelvic Floor Muscle Training

^a^Defined as sedentary or low-intensity physical activity, with no regular higher-intensity physical activity

^b^There were no daily smokers among the study participants, only weekly smokers

^c^Defined as any university education, regardless of duration or completion

^d^eHealth Literacy Scale, an eight-item scale with a score ranging from 8 to 40 points. The scale is used to assess an individual’s ability to identify, evaluate, and use eHealth resources, with higher scores indicating a stronger ability

^e^The conditions included heart disease, asthma, depression, anxiety, renal disease, cancer, and other (not specified)

^f^Note that the use of anticholinergic medications was an exclusion criterion in the study

^g^The degree of constipation among participants ranged from occasionally constipated to constantly constipated

^h^A higher score reflects more severe symptoms

^i^The number of urinary leakages per week, calculated from a two-day bladder diary

^j^Participants allocated to the control group in the original RCT study received the treatment app after 15 weeks

^k^Participants were asked whether they were confident in their ability to correctly perform a pelvic floor contraction

^l^Participants were asked to rate their level of motivation between 0 (not at all motivated) and 10 (thoroughly motivated)

### Lost to follow-up

Of the original 123 women, the 25 women who were lost to follow-up did not differ from those included in terms of age, education level, type of incontinence, and incontinence symptoms. The only difference was found in the BMI, where those included had a higher mean BMI (mean difference 2.8, 95% CI 1.5–4.2).

### Treatment satisfaction

Of the 98 women, 57 (58%) were satisfied with the long-term treatment effect (Fig. 2). The improvements in all scores from baseline to long-term follow-up were significantly larger in the group that was satisfied in comparison with the group that was not satisfied (*p* < 0.001) (Table 2).

**Table 2:** The outcome variable in relation to change in symptom scores

Variable	Treatment satisfaction at the long-term follow-up			
	Yes *n* = 57	No *n* = 41	Mean difference (95% CI)^a^	*P* value^b^
	Mean (SD)	Mean (SD)		
Change in ICIQ-UI SF score from baseline to long-term follow-up^c^	− 5.58 (3.53)	− 1.93 (3.05)	3.65 (2.30–5.01)	** < 0.001**
Change in ICIQ-OAB score from baseline to long-term follow-up^c^	− 1.74 (1.77)	− 0.56 (1.57)	1.18 (0.49–1.86)	**0.001**
Change in ICIQ-LUTSqol score from baseline to long-term follow-up^c^	− 9.21 (6.70)	− 3.95 (6.54)	5.26 (2.56–7.96)	** < 0.001**

*ICIQ-UI SF* International Consultation on Incontinence Questionnaire (ICIQ) − Urinary Incontinence Short Form, *ICIQ-LUTSqol* ICIQ − Lower Urinary Tract Symptoms Quality of Life Module, *ICIQ-OAB* ICIQ − Overactive Bladder Module

^a^Calculated using Student's *t* test

^b^Bold numbers indicate statistical significance (*P* < 0.05)

^c^Negative change values indicate symptom improvement

### Variables associated with treatment satisfaction

#### Baseline model

The crude analysis showed that the following categorical variables met the criteria for further investigation: treatment expectations, previous healthcare contact, BMI, constipation, other chronic conditions, and concomitant medication. Of the continuous variables, ICIQ-UI SF, ICIQ-LUTSqol, and ICIQ-OAB met the criteria. Due to a significant overlap between the factors measured by these scores, we chose to include only the ICIQ-LUTSqol in the multivariable model.

Most participants were both highly motivated and confident: 92/98 (94%) rated their motivation in the highest quartile of a 0–10 scale, and 82/98 (84%) rated their confidence in the same quartile, and the variation was too low for consideration in the model.

The final baseline model included the ICIQ-LUTSqol, incontinence type, randomization group, concomitant medication, and treatment expectations. In this model, two of the five examined variables achieved statistical significance: the ICIQ-LUTSqol score (OR 0.91, 95% CI 0.58–0.97); and concomitant medication (OR 2.84, 95% CI 1.06–7.58) (Table 3, Model I). The variable concomitant medication changed from *p* = 0.086 in the univariate analysis to *p* = 0.037 in the final model.

**Table 3:** Results of the regression models

Model I Baseline factors associated with treatment satisfaction at long-term follow-up						
Baseline variable	Crude OR	95% CI	*P* value^a^	Multi-variable model OR	95% CI	*P* value^a^
ICIQ-LUTSqol score^b^	0.93	0.88–0.98	**0.007**	0.91	0.85–0.97	**0.003**
Incontinence type						
UUI	Ref.	Ref.	0.235	Ref.	Ref.	0.251
MUI	1.74	0.70–4.35		1.88	0.64–5.54	
Treatment access^c^						
Immediate	Ref.	Ref.	0.208	Ref.	Ref.	0.303
Delayed	1.68	0.75–3.79		1.62	0.65–4.03	
Concomitant medication^d^						
No	Ref.	Ref.	0.087	Ref.	Ref.	**0.037**
Yes	2.13	0.90–5.07		2.84	1.06–7.58	
Treatment expectations						
A little or much improved	Ref.	Ref.	0.083	Ref.	Ref.	0.148
Very much improved or completely cured	0.484	0.21–1.10		0.51	0.20–1.27	

Model II Short-term follow-up factors associated with treatment satisfaction at long-term follow-up						
Baseline variable	Crude OR	95% CI	*P* value^a^	Multi-variable model OR	95% CI	*P* value^a^
Symptom improvement (ICIQ-UI SF score)^e^	1.15	1.01–1.30	**0.029**	1.04	0.90–1.21	0.591
Improved ability to endure urgency compared with baseline						
No	Ref.	Ref.	**0.001**	Ref.	Ref.	**0.010**
Yes	5.43	2.05–14.35		4.33	1.43–13.12	
Confident in ability to perform correct pelvic floor contraction						
No	Ref.	Ref.	**0.013**	Ref.	Ref.	**0.041**
Yes	3.00	1.26–7.13		2.67	1.04–6.82	
Motivation for maintenance training^f^						
0–5	Ref.	Ref.	0.055	Ref.	Ref.	0.327
6–10	2.45	0.98–6.15		1.67	0.60–4.69	

Results from the univariate analysis represented as crude OR; results from the multivariate analysis represented as multiple variable OR

*ICIQ-LUTSqol* International Consultation on Incontinence Questionnaire (ICIQ) − Lower Urinary Tract Symptoms Quality of Life Module, *UUI* Urgency urinary incontinence, *MUI* Mixed urinary incontinence, *ICIQ-UI SF* International Consultation on Incontinence Questionnaire (ICIQ) − Urinary Incontinence Short Form

^a^Bold numbers indicate statistical significance (*P* < 0.05)

^b^A higher score corresponds to a greater level of impact on incontinence-related quality of life

^c^Delayed = participants randomized to the original control group who received the app after 15 weeks

^d^Regular medication for one or more of the following conditions: heart disease, hypertension, asthma, depression or anxiety; or diuretics, oral estrogens or hormonal intrauterine device

^e^A one-unit increase in the variable corresponds to one unit of improvement (i.e., a one-unit decrease in the actual score at follow-up)

^f^0 = Not at all motivated; 10 = Thoroughly motivated

### Short-term follow-up model

The results of the univariate analysis of the categorical short-term follow-up variables showed that the variables PFMT frequency, motivation for maintenance training, improved PFMT contraction ability, improved ability to endure urgency, and confidence in performing PFMT contractions correctly, matched the criteria.

All continuous short-term follow-up variables analyzed in the univariate analysis generated statistical significance: the ICIQ-UI SF, ICIQ-LUTSqol, and ICIQ-OAB, as well as improvement in the ICIQ-UI SF and ICIQ-LUTSqol scores.

The final model with variables from the short-term follow-up included the change in incontinence symptoms (ICIQ-UI SF score); ability to endure urgency compared with baseline; confidence in performing PFMT contractions; and level of motivation for the maintenance training. Two of the variables in the model showed statistical significance: improvement in the ability to endure urgency (OR 4.33, 95% CI 1.43–13.12), and confidence in PFMT contractions (OR 2.67, 95% CI 1.04﻿–6.82) (Table 3, Model II).

## Discussion

This study investigated factors associated with long-term treatment satisfaction for women using the Tät®II app for urgency-related urinary incontinence. The majority of the women were satisfied with the treatment 15 months after being given access to the treatment app. Women with less impact on their incontinence-related quality of life at baseline were more likely to experience satisfaction. Long-term satisfaction was also more prevalent in women with an increased ability to endure urgency and a stronger level of confidence in how to contract their pelvic floor directly after treatment.

### Treatment satisfaction

Our finding that 58% of the participants were satisfied with their treatment over the long term, is a promising result proving that app-based treatment can be well-received by women with UUI and MUI. Moreover, long-term treatment satisfaction was strongly associated with improvement in all the symptom scores, both in the short term and long term. A patient who is satisfied with the treatment result is less likely to need additional treatment, which is desirable from a clinical point of view, and patient satisfaction has been suggested as an important outcome measure in studies of urgency-predominant urinary incontinence treatments [10]. Our results can be compared with those regarding other treatment alternatives. For example, a study by Goode et. al. comparing tolteridine treatment with or without a behavioral intervention for 307 women with urgency-predominant incontinence, assessed treatment satisfaction at the end of the 10-week treatment period. In total, 46.3% of the participants were completely satisfied with the treatment results, whereas 48.5% were partly satisfied, and 5.1% were not at all satisfied. Those who received the combined treatment were more likely to be completely satisfied than those who received tolteridine alone [11]. In a cross-sectional study of 997 Japanese patients with pharmacological treatment of OAB, only 33.4% of the respondents were satisfied with their treatment [12].

### Association with treatment satisfaction

#### Incontinence-related quality of life

In the present study, the highest rates of satisfaction were found in those with a lower level of impact on their quality of life at baseline. It is known that more severe incontinence can impact the quality of life negatively, and reduce treatment success and satisfaction [2, 12–14]. Our findings are also consistent with the findings of Bolge et.al. which show that patients with a lower level of impact on their health-related quality of life were found to be more satisfied with the pharmacological treatment of their overactive bladder [10]. We did not investigate urgency-related symptoms specifically in our final model due to the overlap with other variables, but the univariate analysis suggested that there could be a connection with long-term satisfaction. This is strengthened by another study that found that urge incontinence symptoms at baseline lowered the odds of treatment satisfaction in women receiving surgical treatment for stress urinary incontinence [15]. Such symptoms could be connected to a higher risk of treatment failure or adverse events, but the finding could also indicate that UUI symptoms overall can affect subsequent satisfaction.

#### Improved abilities

We found an association between improved PFMT abilities and treatment satisfaction. Encouragingly, an improved ability after 15 weeks was still influential 1 year later. Increased self-efficacy was previously described as being associated with urinary incontinence treatment success [16]. An improved ability to control the bladder and pelvic floor might increase self-efficacy, and it has been associated with an overall improvement in urinary incontinence symptoms [17]. It has also been suggested that women who improve their pelvic floor function during treatment are more likely to experience treatment success than women who already have good pelvic floor control at baseline [18].

### Study population

The original study excluded patients with common chronic conditions such as diabetes, stroke, and multiple sclerosis, thus perhaps selecting a sample that is less burdened by multimorbidity than a general population. Multimorbidity and/or polypharmacy often have a negative impact on multiple health-related aspects [19, 20]. For UUI specifically, it has been suggested that comorbidity affects the burden of disease as well as quality of life [21]. Therefore, the finding that participants with concomitant medication were more likely to experience treatment satisfaction needs to be interpreted with caution. For comparison, in our study population the use of antidepressants or anxiolytic medication was lower than in the general population (11% compared to 16% of Swedish women aged 35–79 years) whereas the use of medication related to heart conditions and/or hypertension was largely similar (16% in our study used medication for these conditions, compared to 13% using beta blockers, the most common heart medication, in the corresponding population) [22].

### Strengths

The RCT that provided the data for this study was registered on ClinicalTrials.org and monitored for proper research conduct; and the development of the app Tät®II incorporated the experience of clinical specialists, and scientific evidence [5]. The loss to follow-up in this study, 25/123 (20%) women, was comparatively small for a 15-month follow-up and data collection was complete for all of those who were included in this present study. Other long-term studies of similar interventions report a rate of between 0 and 39% of losses to follow-up [23–26].

A variety of relevant variables were systematically examined for association with treatment satisfaction and we constructed two separate models to distinguish baseline factors from factors present immediately after the initial 15-week treatment.

The outcome, treatment satisfaction, was selected for its clinical relevance while it also reflected an improvement in symptoms measured across three different validated incontinence symptom scores. The question regarding treatment satisfaction includes the patient’s subjective treatment-related satisfaction, and in contrast to the PGI-I, it is not affected by recall bias, since it measures present satisfaction.

### Limitations

This was a secondary analysis of data and thus the study was not ideally powered to discover weaker associations between variables. The sample size was limited to 98 cases, and we therefore only included 5 variables in each model. There is a chance that a study with a larger population would identify other factors of significance for treatment satisfaction.

The question on treatment satisfaction has not been validated, and the three response options (yes no symptoms, yes with symptoms, or no) did not include any grades.

Some of the variables examined for association with the outcome might have been affected by recall bias; others were conflated due to small groups, potentially resulting in rougher estimations. We endeavored to avoid overlap in the selection of variables to include in the multivariable models, but there remains a chance of unexplained interaction.

The population was homogenous in terms of socioeconomy and the results may not be generalizable to all women with UUI or MUI.

### Clinical implications and future research

A mobile app can be a first-line treatment alternative for clinicians that have female patients with UUI and MUI, as it is associated with a high rate of satisfaction among the users. Both bladder training and pelvic floor contraction ability are important for long-term satisfaction. The clinician might consider offering extra resources to patients who struggle with performing the contractions correctly. One focus of future research should be how to make mobile apps for UUI and MUI more widely available to patients outside the research realm.

## Conclusions

In this study of an app-based treatment for UUI and MUI, the majority of women experienced long-term satisfaction with the treatment. Women with a lower level of incontinence-related impact on their quality of life at baseline were more likely to express long-term satisfaction. Other clinically relevant factors related to treatment satisfaction were the ability to control the bladder and confidence in how to perform pelvic floor contractions immediately after the 15-week treatment. For the clinician, our findings thus suggest that app-based bladder training and PFMT support can be a satisfactory treatment alternative for female patients with UUI or MUI.

## Data Availability

The data that support the findings of this study are available on request from the corresponding author. The data are not publicly available due to privacy or ethical restrictions.
